# Supplementary material for: Aromatic lactic acid production by Bifidobacterium longum subsp. infantis is determined by cell density, substrate availability, and pH in vitro
Source: Appl Environ Microbiol. 2026 May 6;92(6):e02511-25. doi: 10.1128/aem.02511-25 (PMC13274450; doi:10.1128/aem.02511-25)
Supplement: Supplemental material — Fig. S1 to S5; Tables S1 to S4. [file aem.02511-25-s0001.docx]

**Supplementary material**

**Supplementary figures**

***
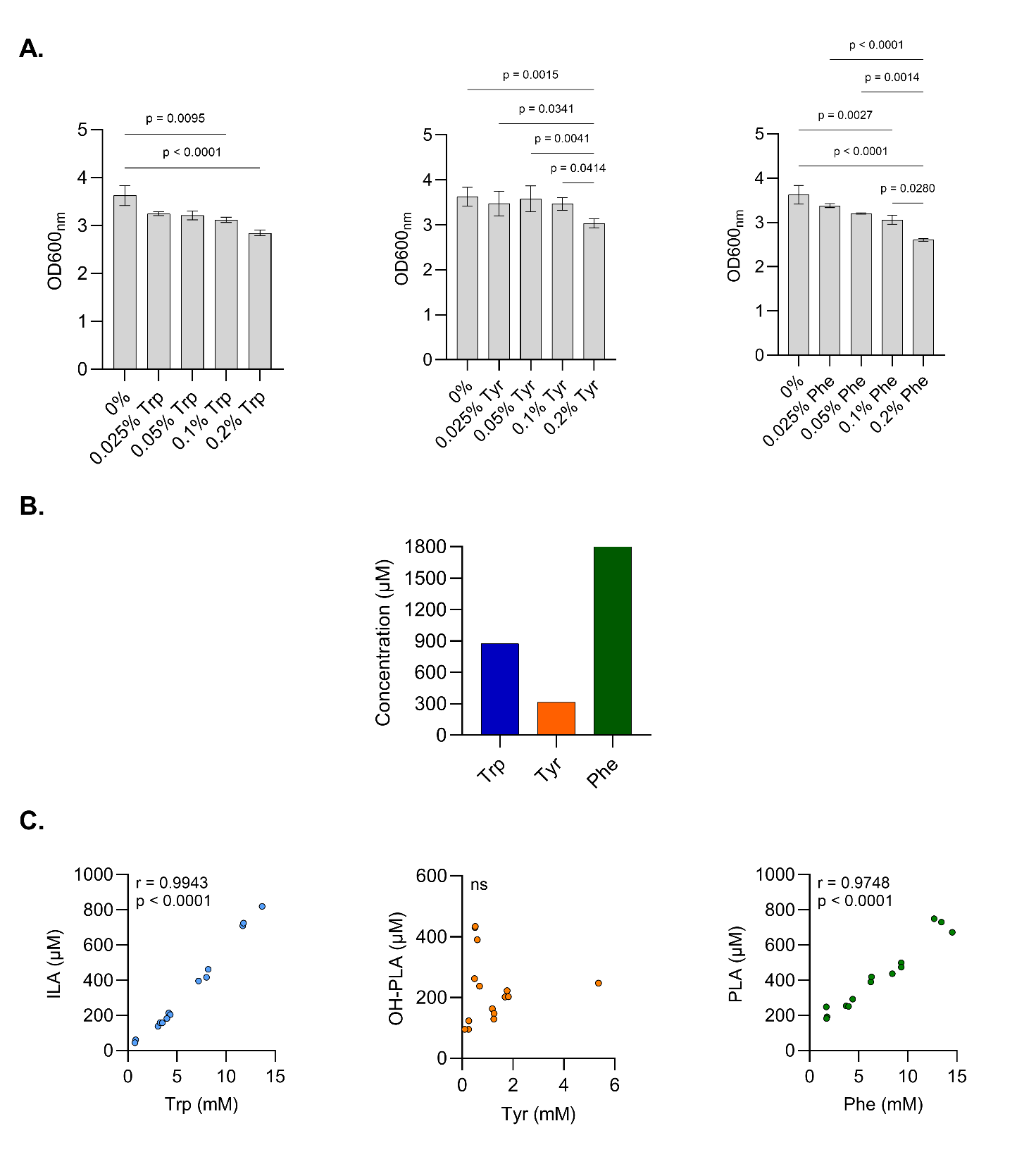
Supplementary Figure 1: (A)*** *Optical density (OD_600nm_) in aromatic amino acid supplemented cultures after 48h of growth in MRS-cys media. Bars show mean ± s.d. of three biological replicates. Statistical significance was evaluated by one-way ANOVA followed by Tukey's multiple comparisons test with adjusted p-values shown on the figure.* ***(B)*** *Correlations between measured concentrations of each aromatic amino acid and their respective aromatic lactic (μM) with Pearson correlation coefficients (r) and p-values (two-tailed) shown in the figures. Points (n = 15) show a single biological replicate.* ***(C)*** *Aromatic amino acid concentrations in non-supplemented MRS-cys broth. Abbreviations: ILA = indole-3-lactic acid; PLA = Phenyllactic acid; OH-PLA = 4-hydroxyphenyllactic acid; Trp = tryptophan; Phe = phenylalanine; Tyr = tyrosine.*


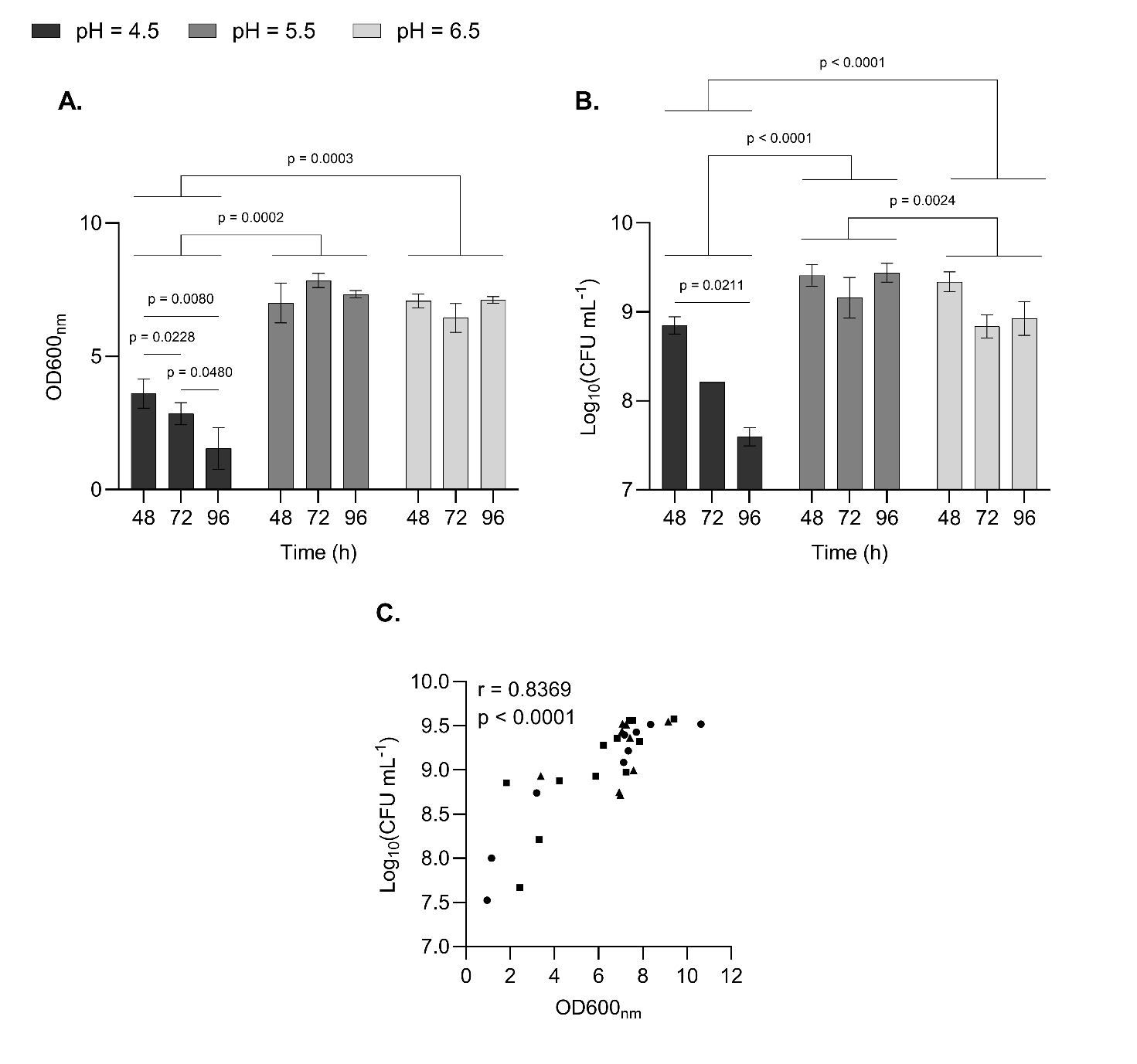


***Supplementary Figure 2: Growth in chemostat cultures. (A)*** *Optical densities (OD_600nm_) at steady state (48h-96h). Bars show mean ± s.d. of three biological replicates. Statistical significance was evaluated by two-way ANOVA followed by Tukey's multiple comparisons test with p-values and adjusted p-values shown on the figure.* ***(B)*** *CFU counts (log-transformed CFU mL⁻¹) at steady state (48h-96h). Bars show the mean ± s.d. of one to three biological replicates. Statistical significance was evaluated by mixed-effects analysis followed by Tukey's multiple comparisons test with p-values and adjusted p-values shown on the figure.* ***(C)*** *Pearson correlation between optical density and CFU counts in chemostat cultures (n = 30), with the Pearson correlation coefficient (r) and p-value (two-tailed) shown on the figure. Biological replicates across pH levels are shown as ▲,* ▮ *and ●.*

*
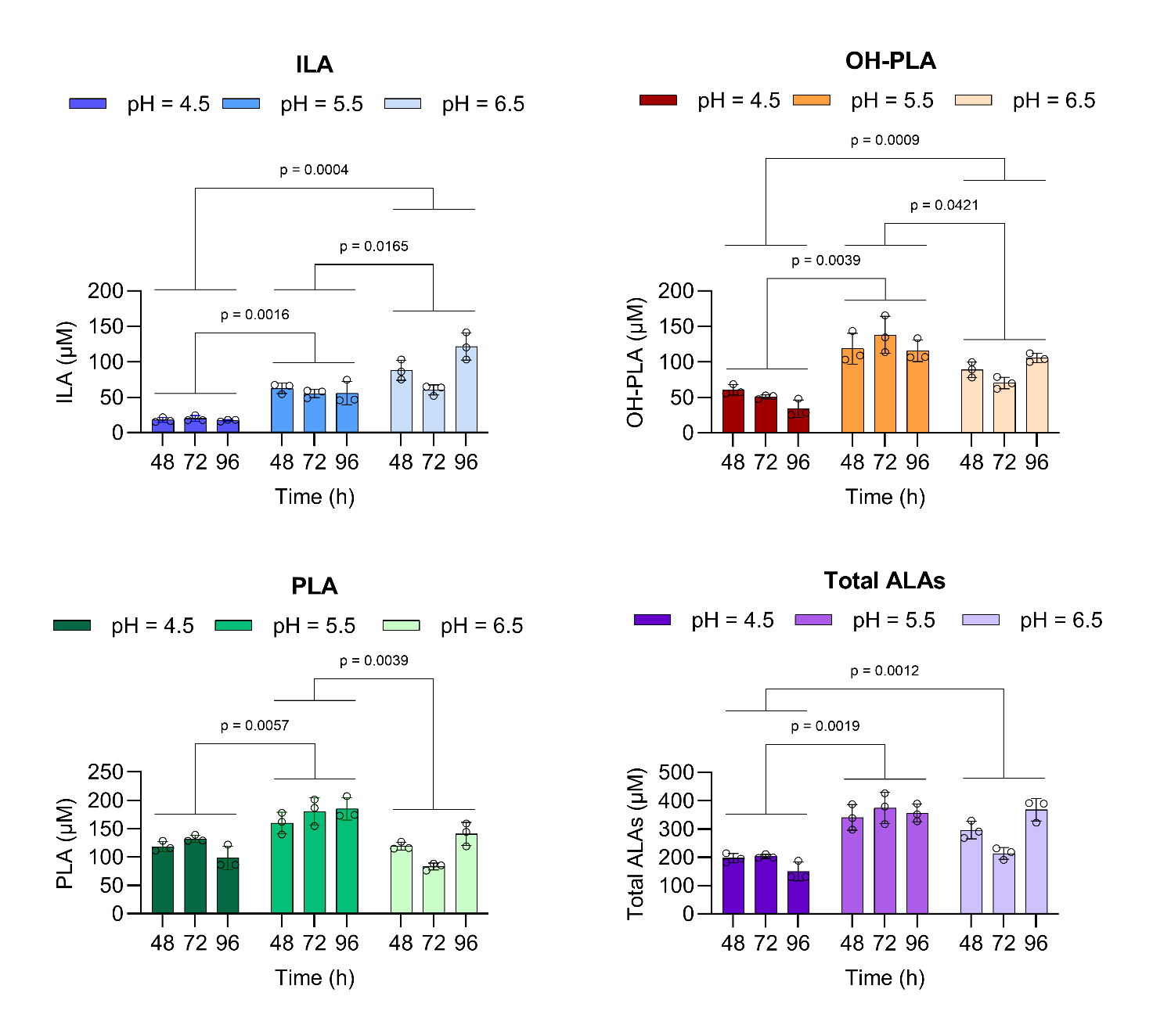
*

***Supplementary Figure 3: Absolute ALA concentrations at different pH levels during steady state.*** *Bars show mean ± s.d. of three biological replicates. Statistical significance between groups were evaluated with two-way ANOVA with p-values of the pH effect shown on the figures. Abbreviations: ILA = indole-3-lactic acid; PLA = Phenyllactic acid; OH-PLA = 4-hydroxyphenyllactic acid; ALAs = aromatic lactic acids.*


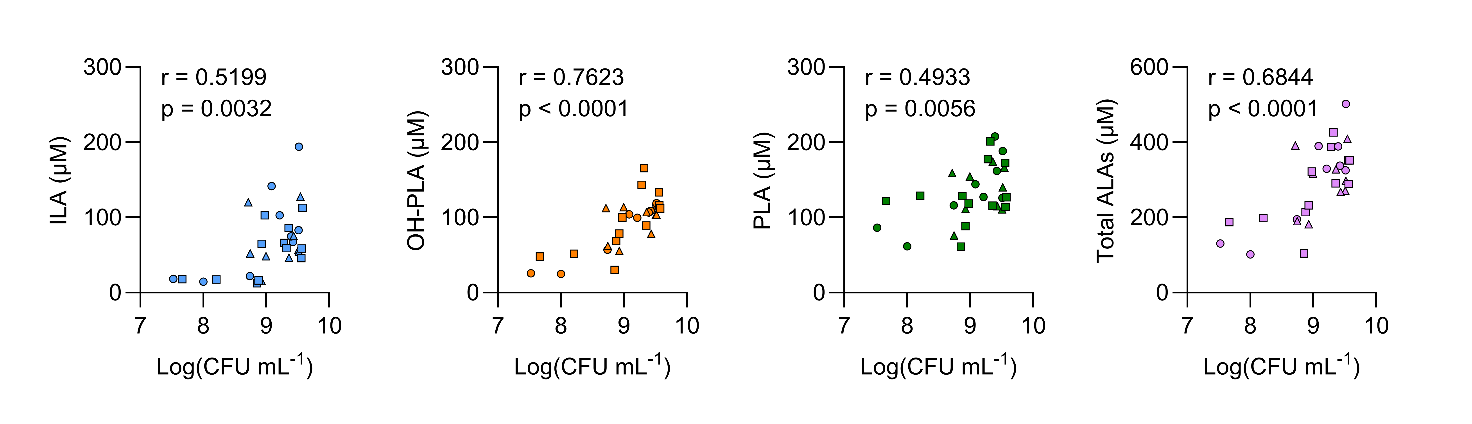


***Supplementary Figure 4: Correlations between CFU counts and ALA concentrations.*** *Correlations (n = 30) between CFU counts (Log(CFU mL^-1^)) and ALAs (μM) in chemostat cultures at 24-96 h with Pearson correlation coefficients (r) and p-values (two-tailed) shown in the figures. Abbreviations: ILA = indole-3-lactic acid; PLA = Phenyllactic acid; OH-PLA = 4-hydroxyphenyllactic acid; ALAs = aromatic lactic acids. Biological replicates are shown as ▲,* ▮ *and ●.*

*
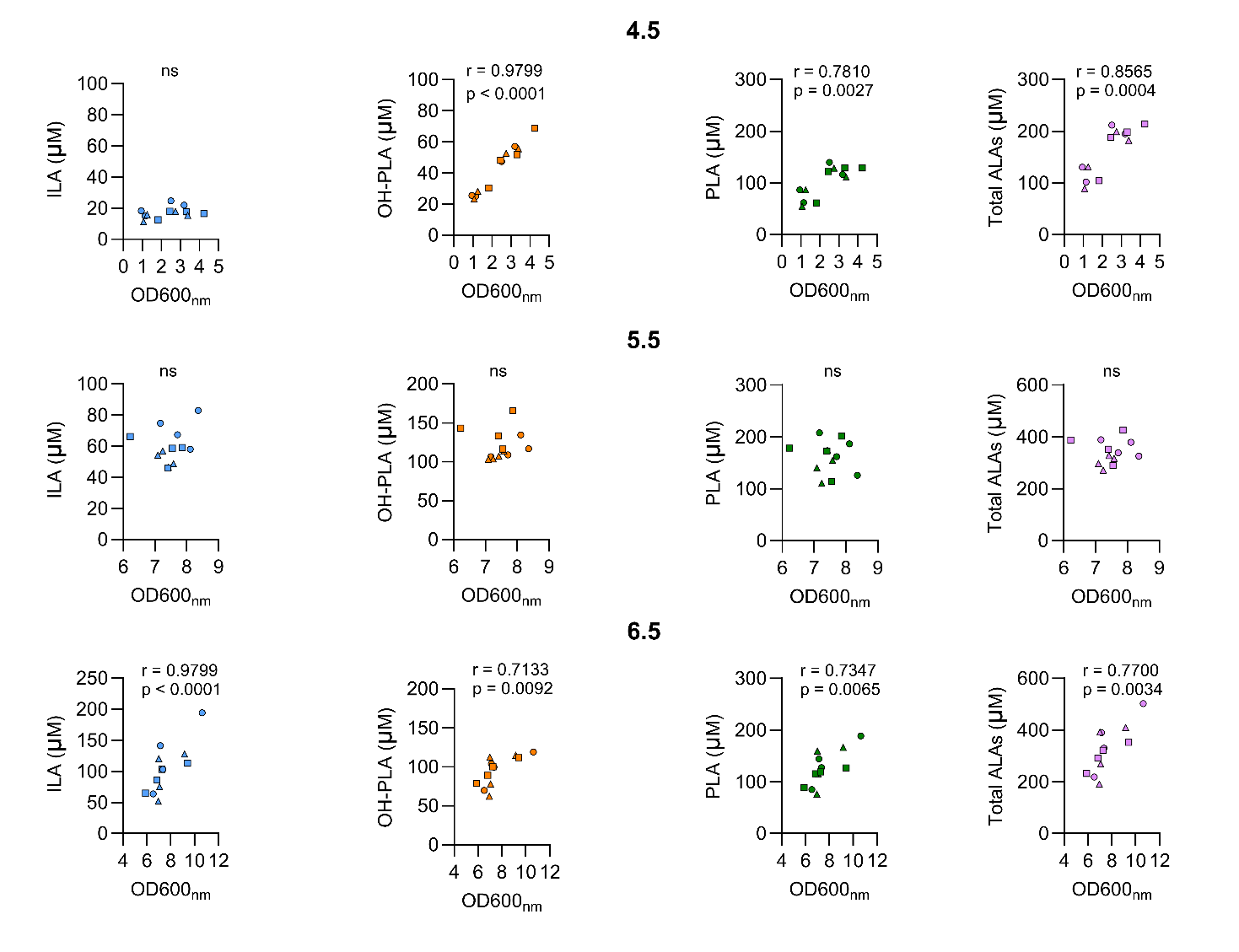
*

***Supplementary Figure 5: Correlations between optical density and aromatic lactic acid production at each pH condition.*** *Pearson correlation between optical density (OD_600nm_) and aromatic lactic acid concentrations in chemostat cultures at each pH level (n = 12), with the Pearson correlation coefficient (r) and p-value (two-tailed) shown on the figures.* *Biological replicates are shown as ▲,* ▮ *and ●.*

**Supplementary tables**

| Pathway | Compound | Retention time (min) | m/z (±0.02) |
| --- | --- | --- | --- |
| Phenylalanine | Phenylalanine (Phe) | 2.7 | 164.0706 |
|  | Phenyllactic acid (PLA) | 5.3 | 165.0546 |
| Tryptophan | Tryptophan (Trp) | 3.9 | 203.0815 |
|  | Indole-3-lactic acid (ILA) | 5.5 | 204.0655 |
| Tyrosine | Tyrosine (Tyr) | 1.3 | 180.0655 |
|  | 4-Hydroxyphenyllactic acid (OH-PLA) | 4.0 | 181.0495 |
| Internal standard | L-Tryptophan (indole-d5) | 3.9 | 208.1129 |

***Supplementary Table 1: Analytes used for LC-MS with observed retention times and m/z in negative ionization mode.***

| **Gene name** | **Gene ID** | **Primer direction** | **Sequence (5’-3’)** | **Tm (°C)** | **GC%** | **Product size (bp)** | **Primer efficiency (%)** |
| --- | --- | --- | --- | --- | --- | --- | --- |
| *aldh* | BLON_RS05510 | Forward | cgacgtgatggcttggtatg | 59 | 55 | 110 | 101.3 |
|  |  | Reverse | cttcgccgatgatggtcttg | 59 | 55 |  |  |
| *pdxS* | BLON_RS10355 | Forward | actcaaccgtaatctggccc | 60 | 55 | 196 | 102.3 |
|  |  | Reverse | agcttcctgaatgcccttga | 59 | 50 |  |  |
| *gluC* | BLON_RS03600 | Forward | cttctcggccactatgacgt | 60 | 55 | 161 | 99.8 |
|  |  | *Reverse* | *aacagttccacatatgcgcc* | *59* | *50* |  |  |
|  |  | *Reverse* | *tcgtccttctcctcgttacg* | *59* | *55* |  |  |

***Supplementary Table 2: RT-qPCR primer information and sequences.***

| ***Sample*** | ***Total Raw Reads*** | ***Total Non rRNA Reads*** | ***Total HQ***  ***Reads*** | ***HQ Bases***  ***(Q30)*** | ***GC Content*** | ***rRNA Reads***  ***%*** | ***HQ Reads***  ***%*** |
| --- | --- | --- | --- | --- | --- | --- | --- |
| *pH 4.5 T48* | *36 M* | *25.22 M* | *24.78 M* | *97.48%* | *60.72%* | *29.94%* | *68.83%* |
| *pH 4.5 T48* | *30 M* | *22.04 M* | *21.78 M* | *96.43%* | *60.68%* | *26.53%* | *72.58%* |
| *pH 4.5 T48* | *36 M* | *23.63 M* | *23.34 M* | *97.37%* | *60.70%* | *34.35%* | *64.83%* |
| *pH 5.5 T48* | *36 M* | *24.03 M* | *23.3 M* | *97.40%* | *59.52%* | *33.25%* | *64.73%* |
| *pH 5.5 T48* | *36 M* | *25.13 M* | *24.4 M* | *96.34%* | *59.94%* | *30.20%* | *67.79%* |
| *pH 5.5 T48* | *36 M* | *25.79 M* | *25.1 M* | *96.36%* | *59.33%* | *28.35%* | *69.71%* |
| *pH 6.5 T48* | *36 M* | *28.23 M* | *27.8 M* | *97.57%* | *60.74%* | *21.59%* | *77.21%* |
| *pH 6.5 T48* | *36 M* | *20.02 M* | *19.54 M* | *97.62%* | *59.61%* | *44.38%* | *54.27%* |
| *pH 6.5 T48* | *36 M* | *26.63 M* | *26.13 M* | *97.51%* | *60.37%* | *26.04%* | *72.59%* |
| *pH 4.5 T72* | *30.07 M* | *19.39 M* | *18.95 M* | *96.26%* | *60.63%* | *35.54%* | *63.02%* |
| *pH 4.5 T72* | *36 M* | *26.03 M* | *25.51 M* | *96.50%* | *60.62%* | *27.70%* | *70.85%* |
| *pH 4.5 T72* | *36 M* | *20.02 M* | *19.49 M* | *96.19%* | *60.65%* | *44.38%* | *54.14%* |
| *pH 5.5 T72* | *36 M* | *25.79 M* | *25.1 M* | *97.43%* | *59.55%* | *28.36%* | *69.71%* |
| *pH 5.5 T72* | *36 M* | *28.22 M* | *27.57 M* | *96.61%* | *59.55%* | *21.60%* | *76.58%* |
| *pH 5.5 T72* | *36 M* | *28.2 M* | *27.52 M* | *96.51%* | *59.60%* | *21.68%* | *76.45%* |
| *pH 6.5 T72* | *36 M* | *23.16 M* | *22.6 M* | *97.66%* | *59.25%* | *35.66%* | *62.78%* |
| *pH 6.5 T72* | *36 M* | *24.54 M* | *24.24 M* | *97.53%* | *60.22%* | *31.84%* | *67.34%* |
| *pH 6.5 T72* | *36 M* | *26.77 M* | *26.36 M* | *97.41%* | *60.84%* | *25.64%* | *73.23%* |
| *pH 4.5 T96* | *31.35 M* | *23.41 M* | *23.11 M* | *96.36%* | *59.06%* | *25.34%* | *73.72%* |
| *pH 4.5 T96* | *36 M* | *23.08 M* | *22.68 M* | *97.42%* | *60.42%* | *35.90%* | *62.99%* |
| *pH 4.5 T96* | *36 M* | *26.84 M* | *26.46 M* | *97.61%* | *60.72%* | *25.44%* | *73.49%* |
| *pH 5.5 T96* | *36 M* | *26.33 M* | *25.83 M* | *96.58%* | *59.92%* | *26.87%* | *71.74%* |
| *pH 5.5 T96* | *36 M* | *29 M* | *28.37 M* | *97.50%* | *58.28%* | *19.46%* | *78.80%* |
| *pH 5.5 T96* | *36 M* | *28.41 M* | *27.84 M* | *97.63%* | *60.11%* | *21.09%* | *77.33%* |
| *pH 6.5 T96* | *36 M* | *30.19 M* | *29.72 M* | *97.54%* | *61.33%* | *16.14%* | *82.56%* |
| *pH 6.5 T96* | *36 M* | *30.99 M* | *30.54 M* | *97.38%* | *60.93%* | *13.93%* | *84.83%* |
| *pH 6.5 T96* | *36 M* | *25.23 M* | *24.71 M* | *97.52%* | *60.65%* | *29.91%* | *68.64%* |

***Supplementary Table 3. RNA-seq sequencing and quality control metrics for all samples.*** *For each sample, the following QC metrics are provided: Sample Name, Total Raw Reads (the total number of raw sequencing reads generated for the sample), Total non-rRNA Reads (the total number of reads after removal of rRNA reads), Total HQ Reads (the total number of high quality reads after sequence cleaning and filtering), HQ Bases (Q30) (Percentage of high quality bases having at least phred quality 30), GC Content (GC content in percentile of high quality sequencing reads), rRNA Reads % (rRNA Reads percentage) and HQ Reads % (High Quality Reads percentage).*

|  | | **pH 4.5 vs 5.5** | | | **pH 4.5 vs 6.5** | | | **pH 5.5 vs 6.5** | | |
| --- | --- | --- | --- | --- | --- | --- | --- | --- | --- | --- |
| **Gene name** | **Gene ID** | **48 h** | **72 h** | **96 h** | **48 h** | **72 h** | **96 h** | **48 h** | **72 h** | **96 h** |
| *Type I glutamate-ammonia ligase* | BLON_RS09840 | 1.57 ***** | 1.75 ***** | 1.49 ***** | 2.46 ***** | 2.37 ***** | 2.38 ***** | 0.89 ***** | 0.62 | 0.89 ***** |
| *Ketol-acid reductoisomerase* | BLON_RS00695 | 2.16 ***** | 2.23 ***** | 1.98 ***** | 5.05 ***** | 4.89 ***** | 4.72 ***** | 2.89 ***** | 2.66 ***** | 2.74 ***** |
| *F_0_F_1_-ATPase genes* | BLON_RS01575 | 0.85 ***** | 1.34 ***** | 1.00 ***** | 0.92 ***** | 1.24 ***** | 2.22 ***** | 0.08 | -0.10 | 1.21 ***** |
|  | BLON_RS01580 | 1.24 ***** | 1.24 ***** | 0.96 ***** | 1.32 ***** | 1.24 ***** | 2.54 ***** | 0.08 | -0.01 | 1.58 ***** |
|  | BLON_RS01585 | 1.04 ***** | 1.23 ***** | 0.98 ***** | 1.29 ***** | 1.31 ***** | 2.39 ***** | 0.25 | 0.08 | 1.41 ***** |
|  | BLON_RS01590 | 1.66 ***** | 1.55 ***** | 1.08 * | 1.89 ***** | 1.63 ***** | 2.84 ***** | 0.24 | 0.08 | 1.76 * |
|  | BLON_RS01595  BLON_RS01600  BLON_RS01605 | 1.38 *****  1.37 *****  1.50 ***** | 1.36 *****  1.15 *****  1.07 ***** | 0.86 *****  0.75  0.67 | 2.10 *****  1.95 *****  1.97 ***** | 1.66 *****  1.53 *****  1.46 ***** | 2.77 *****  2.66 *****  2.57 ***** | 0.71  0.58  0.47 | 0.30  0.39  0.39 | 1.91 *****  1.90 *****  1.90 ***** |
|  |  |  |  |  |  |  |  |  |  |  |
| *dnaK* | BLON_RS00740 | 1.30 ***** | 1.32 ***** | 0.57 | 2.28 ***** | 1.71 ***** | 2.33 ***** | 0.96 * | 0.39 | 1.76 ***** |
|  |  |  |  |  |  |  |  |  |  |  |
| *GroEl* | BLON_RS03520 | 1.71 * | 1.86 ***** | 1.01 ***** | 2.98 ***** | 3.10 ***** | 3.52 ***** | 1.21 * | 1.24 ***** | 2.51 ***** |
| *GroES* | BLON_RS11670 | 1.29 ***** | 1.60 ***** | 0.97 ***** | 3.00 | 3.58 ***** | 3.86 ***** | 1.72 ***** | 1.97 ***** | 2.90 ***** |

***Table 4: Differential expression of stress response genes in Bifidobacterium.*** *The table shows log₂ fold changes at each time point, calculated from three biological replicates. *: Genes with an average read count greater than 100 across all samples and an FDR-corrected p-value < 0.1 for the log₂ fold change are considered significant.*
